# Ribosomes in the balance: structural equilibrium ensures translational fidelity and proper gene expression

**DOI:** 10.1093/nar/gku1020

**Published:** 2014-11-11

**Authors:** Sharmishtha Musalgaonkar, Christine A. Moomau, Jonathan D. Dinman

**Affiliations:** Department of Cell Biology and Molecular Genetics, University of Maryland, College Park, MD 20742, USA

## Abstract

At equilibrium, empty ribosomes freely transit between the rotated and un-rotated states. In the cell, the binding of two translation elongation factors to the same general region of the ribosome stabilizes one state over the other. These stabilized states are resolved by expenditure of energy in the form of GTP hydrolysis. A prior study employing mutants of a late assembling peripheral ribosomal protein suggested that ribosome rotational status determines its affinity for elongation factors, and hence translational fidelity and gene expression. Here, mutants of the early assembling integral ribosomal protein uL2 are used to test the generality of this hypothesis. rRNA structure probing analyses reveal that mutations in the uL2 B7b bridge region shift the equilibrium toward the rotated state, propagating rRNA structural changes to all of the functional centers of ribosome. Structural disequilibrium unbalances ribosome biochemically: rotated ribosomes favor binding of the eEF2 translocase and disfavor that of the elongation ternary complex. This manifests as specific translational fidelity defects, impacting the expression of genes involved in telomere maintenance. A model is presented describing how cyclic intersubunit rotation ensures the unidirectionality of translational elongation, and how perturbation of rotational equilibrium affects specific aspects of translational fidelity and cellular gene expression.

## INTRODUCTION

Successful conversion of genetic information from mRNA to proteins requires efficient and accurate functioning of the highly orchestrated nanomachine called the ribosome, a complex ribonucleoprotein particle universally composed of two subunits. Yeast ribosomes contain approximately 80 proteins and 4 rRNA molecules (1,2). Its high level of structural complexity confers the flexibility and versatility required to interact with a wide range of *trans*-acting ligands (3,4). These include aminoacyl-tRNA containing ternary complexes (TC), the eEF2 translocase, and a host of release and recycling factors. The unidirectionality of translation elongation is energetically driven by several guanosinetriphosphate (GTP) hydrolysis reactions coupled to the preferential binding of two intersubunit rotational states with two structurally similar ligands (3–7). The two extreme stages of ribosomal rotation are called unrotated and rotated states (8,9). It appears that intersubunit rotation is also accompanied by intersubunit ‘rolling’ motions in eukaryotes (10). During the translation elongation cycle, some intersubunit bridges function as ‘pivot points’ upon which intersubunit rotation is balanced, while others are transient, breaking and re-forming as the subunits move between the two states. The B3 intersubunit bridge is an example of a pivot, while the B7a bridge exemplifies a rotational state-dependent interaction (11,12). Eukaryotic ribosomes contain more intersubunit bridges than eubacterial or archaeal ribosomes, most of which comprise protein–protein interactions (13). At equilibrium, empty ribosomes can freely rotate among as many as 40–50 conformations (11,14). Studies of yeast uL16 (previously known as L10) (15) suggested that unbalancing this rotational equilibrium perturbs allosteric communication pathways within the ribosome. These in turn affect the steady-state affinities for *trans*-acting factors, which manifest themselves as changes in translational fidelity (16). Programmed alterations in translational fidelity have recently been shown to be responsible for regulating the expression of specific genes in from yeast to humans (17–19), and global changes in translational fidelity, in particular in programmed -1 ribosomal frameshifting, have been linked to at least three human diseases (20–22).uL16 is assembled at the end of the LSU maturation process, is located on the periphery of the LSU and directly interacts with the elongation factors (23). A correlation has been noted between defects in peripheral/late assembling ribosome proteins and a class of human diseases collectively known as ribosomopathies (22). To address the question of whether the connection between rotational equilibrium and translational fidelity is specific to this class of ribosomal proteins or if it is a more generalized phenomenon, we tested it using mutants of the universally conserved core ribosomal protein uL2 (previously known as L2) (15). uL2 is incorporated into ribosomes in the early stages of biogenesis (24). Structurally, uL2 contains a solvent-accessible globular domain that interfaces with the small subunit (SSU) through intersubunit bridge B7b (Supplementary Figure S1). This is linked to a second domain that closely approaches the peptidyltransferase center through a functionally important ‘neck’. uL2 is an integral protein that interacts with nearly every domain of the large subunit (LSU) rRNA (13,25). Along with uL3, uL2 is required to maintain the peptidyltransferase center, and indeed, early ribosome reconstitution studies suggested that histidine residues in the basic extension domain might directly participate in the peptidyltransferase reaction (24). Mutants of uL2 have been characterized in both yeast and *Escherichia coli* demonstrating conserved roles in subunit association and in ribosome structure, biochemistry and translational fidelity (26,27). In the current study, mutants of uL2 located in the SSU interface region were used to test the model of the importance of maintaining intersubunit rotational equilibrium on ribosome function. Consistent with prior studies of uL16, mutations of uL2 that drive the equilibrium toward the rotated state promote allosteric changes in functional centers of both subunits that favor binding of eEF2 and disfavor that of TC. These in turn manifest as specific alterations in translational fidelity, which are biologically manifested, in part, by decreased telomere length. A model describing how this perturbation of ribosome structural equilibrium alters specific aspects of translational fidelity is presented.

## MATERIALS AND METHODS

### Strain plasmids and media and generation of *rpL2A* mutants

*Saccharomyces cerevisiae* strain yJD1269 expressing wild-type *RPL2A* from a centromeric *URA3* vector (pRPL2A-URA3) was previously described (26). In yJD1315, wild-type *RPL2A* is supported by a centromeric *LEU2* vector (pRPL2A-LEU2 or pJD957). Mutations in the *RPL2A* ORF of pRPL2A-*LEU2* were generated by oligonucleotide site-directed mutagenesis using the Quikchange XL site-directed mutagenesis kit (Stratagene, Madison, WI, USA). Plasmids were amplified in *E. coli* strain DH5α. Mutant *rpl2A* strains were generated by replacing pRPL2A-URA3 with pRPL2Amut-LEU2 through standard 5-FOA plasmid shuffle technique (29). *Escherichia coli* were transformed using the calcium chloride method (30) and yeast cells were transformed by the alkali cation protocol (31). YPAD, synthetic complete (SC), synthetic dropout medium (H-) and 4.7 MB plates for testing the killer phenotype were used as previously described (32,33). The *S. cerevisiae* strains and oligonucleotides (IDT, Coralville, IA, USA) used in this study are shown in Supplementary Tables S1 and S2.

### Growth and translation fidelity assays

Dilution spot assays were used to qualitatively monitor cell growth at 30, 37 and 15°C. For all conditions, yeast cells were grown to logarithmic growth phase and then diluted to 10^6^ CFU/ml. Subsequently, 10-fold serial dilutions of each strain were spotted onto YPAD. The dual luciferase reporter plasmids pYDL-control, pYDL-LA, pYDL-Ty*1*, pYDL-UAA (34), pYDL-AGC_218_ and pYDL-TCT_218_ (35) were employed to monitor L-A-directed programmed -1 ribosomal frameshifting (-1 PRF), Ty*1*-directed +1 PRF, UAA termination codon readthrough and suppression of an AGC near-cognate serine codon or a TCT noncognate serine codon in the firefly luciferase catalytic site, respectively. All assays were performed in quadruplicate and were repeated a minimum of four times or until the statistical requirements were met (36). Sample readings were collected using a GloMax Multi-Microplate luminometer (Promega).

### Biochemistry of ribosomes and *trans*-acting factors

Ribosomes were purified as previously described (16) with a final modification involving removal of residual salt from re-suspended ribosomes by dialysis against low-salt storage buffer [20 mM HEPES-KOH pH 7.6, 50 mM NH_4_Cl, 5 mM Mg(OAc)_2_, 1 mM DTT, 25% glycerol]. Recombinant His-tagged yeast phenyalanyl-tRNA synthetase (yPheRS) was expressed in *E. coli* using an inducible system (37). pQE32- expressing the alpha and beta subunits of yPheRS as a fusion protein yPheRS (a generous gift from Dr David Tirrell, California Institute of Technology) was transformed into *E. coli* strain SG13009 [pREP4] (Qiagen). Transformed cells were grown in 2xYT media containing 100 μg/ml carbenicillin and 35 μg/ml kanamycin to 0.6 O.D._595_ and protein expression was induced with 1 mM isopropyl-β-D-thiogalactopyranoside (IPTG). Cells were harvested after 4 h and the His-tagged protein was purified over nickel-NTA agarose affinity purification resin under native condition according to manufacturer's protocol (Qiagen). Imidazole and high salt in the elution buffer were removed by dialyzing against storage buffer (50 mM Tris-HCl, pH 7.5, and 1 mM DTT) using Amicon ultra 50 kDa columns. The 3′-terminal CCA ends of yeast tRNA^Phe^ (obtained from Sigma-Aldrich) were repaired in reactions containing 20 mM Glycine-NaOH, pH 9.0, 10 mM MgCl_2_, 16 μM tRNA, 160 μM CTP, 160 μM ATP using tRNA terminal nucleotidyltransferase purified from yeast by gel filtration and ion-exchange chromatography (300 mM NaCl fraction) of whole cell yeast lysates (38). Repaired tRNA was purified by phenol extraction followed by ethanol precipitation. Yeast tRNA^Phe^ was aminoacylated in a 4 ml reaction mixture containing 16 μM tRNA^Phe^, 40 μM [^14^C]Phe, 10 mM ATP, 100 mM HEPES-KOH (pH 7.6), 9 mg yPheRS (1 AU/250 mg) 10 mM KCl, 20 mM MgCl_2_ and 1 mM DTT. Determination of steady-state-binding constants for tRNAs was performed as previously described with modifications (28,39). Ribosomes were pre-activated in binding buffer containing polyU and deacylated tRNA^Phe^. Ternary complex was preassembled using HPLC-purified [^14^C] Phe-tRNA^Phe^, GTP, and soluble ammonium sulfate fraction containing yeast elongation factors (40). Reactions to monitor binding of TC to the A-site contained constant amounts of ribosomes (33.33 nM) and 2-fold serial dilutions of TC (0–128 pmoles) and filter binding assays were performed as previously described (28). Assays to monitor binding of Ac-[^14^C] Phe-tRNA^Phe^ to the ribosomal P-site were performed using preactivated ribosomes incubated with serial dilutions of HPLC-purified Ac-[^14^C] Phe-tRNA^Phe^ as described (28). 6xHis-tagged eEF2 was purified from TKY675 yeast cells (kindly provided by Dr T. Kinzy) (30) and ribosome-binding assays were performed as described (79) with the sole modification that reactions contained 5 pmols of salt washed 80S ribosomes in 50 μl reaction volumes. Reactions were performed three times in duplicate. K_D_ values were determined assuming single binding sites using Graphpad Prism software.

### rRNA chemical modification and structural analyses

hSHAPE (41) of rRNA with 1M7 was performed as described (42) using the following primers: 969 and 1780 in the SSU, and 25–2, 1466, 2632, 2836, 25–7 and 3225 in the LSU. Briefly, rRNA from chemically treated wild-type and mutant ribosomes was extracted and extension reactions performed using fluorescently labeled primers, followed by fragment analysis using capillary electrophoresis. Data were analyzed using SHAPEFinder (43). For kethoxal studies, 25 pmoles of purified ribosomes in a 50 μl volume were treated with 1 μl of a 4% kethoxal solution (in pure ethanol), or 1 μl of ethanol as control, and incubated for 10 min at 30°C. Reactions were stopped by the addition of one half volume of stop solution (150 mM sodium acetate, 250 mM potassium borate), followed by ethanol extraction and extension reactions were performed as described above using primer 969. Scored data were mapped onto 3.0 Å resolution yeast ribosomes (13) using PyMOL (44).

### mRNA abundance and telomere length analyses

Quantitative PCR experiments to assay mRNA abundance were carried out as described (17). Similar methods were used to quantify telomere length in yeast cells (22). Genomic DNA was isolated from mid-logarithmic cell cultures as previously described (29). Quantitative polymerase chain reactions to determine yeast telomere content (T) relative to the single-copy reference gene SGS1 (S) were performed on serially diluted DNA using the Bio-Rad iTaqTM Universal SYBR Green system utilizing primer pairs complementary to telomere repeats (Supplementary Table S2), and cycle threshold values were determined. The T/S ratios (relative telomere content) were calculated from three experimental replicates at each of three DNA concentrations (100, 200 and 400 ng). The Student's *t*-test for two-tailed *P* value calculations was used throughout.

## RESULTS

### Genetic characterization of mutants in the vicinity of the B7b bridge

Ribosomal protein uL2 is roughly bilobular. One domain approaches the P-site of the peptidyltransferase center (Supplementary Figure S1B) and the other interacts with the small subunit through the B7b intersubunit bridge (Supplementary Figure S1A, S1C). The results of a prior random mutagenesis study suggested that structural flexibility between the two domains may help to coordinate tRNA–ribosome interactions (26). Subsequently, studies of ribosomal protein uL16 suggested that changes in the equilibrium between intersubunit rotational states underlie biochemical, translational fidelity and gene expression defects. The current study focuses on the B7b bridge to further test and expand upon this hypothesis.

Using atomic (13) and near-atomic (45) resolution yeast ribosome structures as a guide, six different regions of uL2 were identified as potentially interacting with the SSU. These are color coded in Supplementary Figure S1. Initially, stretches of up to five amino acids in each region were mutated to alanine. With one exception, none of these were viable as the sole form of uL2 (Supplementary Figure S1D). Subsequently, mutants were made containing one, two or three alanine substitutions and were scored for viability. Dilution spot assays were employed to score the growth phenotypes of the viable mutants at high and low temperatures (Supplementary Figure S2A), ‘Killer’ assays were employed to score the ability of these mutants to maintain the yeast killer virus (Supplementary Figure S2B) and all mutants were assayed with regard to their quantitative effects on four aspects of translational fidelity: -1 PRF, +1 PRF, UAA termination codon readthrough and suppression of a near-cognate codon (Supplementary Figure S2C). From these genetic analyses, the four mutants with the most pronounced phenotypes were selected for further characterization: H139-E143A, uL2-K177A, deletion of the C-terminal end (248–254Δ) and uL2-Y133A.

### uL2 mutants in the B7b intersubunit bridge region disrupt the rotational equilibrium of ribosomes

**Figure 1.**
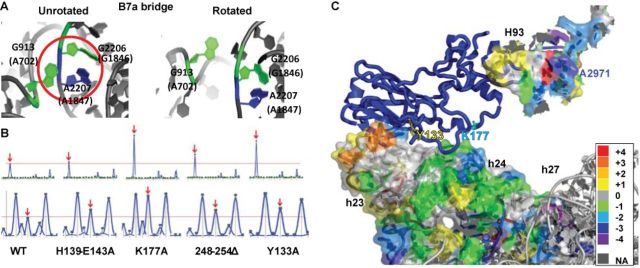
uL2 B7b bridge mutants alter the rotational equilibrium of the ribosome. (**A**) The B7a intersubunit bridge. In the nonrotated ribosome, A2207 (25S rRNA) and G913 (18S rRNA) engage in a triple-base interaction along with G2206. In the rotated state, the base triple is disrupted, and the 2′ OH-group on A2207 becomes accessible to modification by 1M7. Similarly, atoms on G913 become accessible to modification by kethoxal upon rotation. Images were generated in PyMOL using atomic and near atomic resolution yeast ribosomal structures (13,45). *Escherichia coli* base numbers are shown in parentheses. (**B**) Reactivity peaks obtained by hSHAPE after chemical probing of the landmark base G913 (arrows) at the SSU side of the B7a intersubunit bridge with kethoxal (upper panel) and probing of the landmark base A2207 (arrows) at the LSU side of the B7a intersubunit bridge with 1M7 (lower panel). (**C**) Differences in 1M7 reactivity between uL2-K177A and WT 18S and 25S rRNA regions probed by hSHAPE. The reactivity differences were assigned color codes according to the scale shown to the right. Warmer colors indicate increased reactivity and cooler colors denote decreased reactivity. Scored data were mapped on 3.0 Å resolution yeast ribosomes (13) using PyMOL.

A comprehensive analysis using hSHAPE defined the chemical reactivity profiles of nonrotated and rotated yeast ribosomes (16). This study established that the reactivity of the two bases in the B7a intersubunit bridge, G913 to kethoxal and A2207 to 1M7, can be used as diagnostic markers of intersubunit rotational status. The involvement of these two bases in a base triple interaction in the unrotated state render them resistant to chemical modification, while disruption of this interaction in the rotated state allows them to react (Figure 1A). The extent of base chemical modification was measured in bulk equilibrium using isogenic wild-type and mutant ribosomes. The reactivity of these two bases in wild-type ribosomes was used to set the baseline for rotational equilibrium at a steady state. Thus, increases or decreases in base modification are indicative of shifts in equilibrium, rotated or unrotated, respectively. Chemical modification analyses revealed increased extents of chemical modification for all four mutants (Figure 1B), indicating shifts in equilibrium toward the rotated state. The most pronounced shifts were observed for the uL2-K177A and uL2-Y133A mutants. More extensive hSHAPE analyses revealed that the uL2-K177A and uL2-Y133A promoted similar changes in 1M7 base modifications in both the SSU and LSU (Figure 1C and Supplementary Figure S3). In the SSU (Supplementary Figure S3a), these mutants tend to render bases in h23 more susceptible to chemical modification, i.e. more flexible. Conversely the two mutants made bases in h23a, h24 and h27 less prone to modification, i.e. less flexible. In the LSU (Supplementary Figure S3b), these two mutants had very strong effects on the chemical reactivity of bases in H93 in the peptidyltransferase center (also see Figure 1C), Helices 89–92 (the tRNA accommodation corridor), and H69 which interrogates the decoding center during translation elongation.

### Disruption of ribosome rotational equilibrium by uL2 mutants affects binding of translation elongation factors

**Figure 2.**
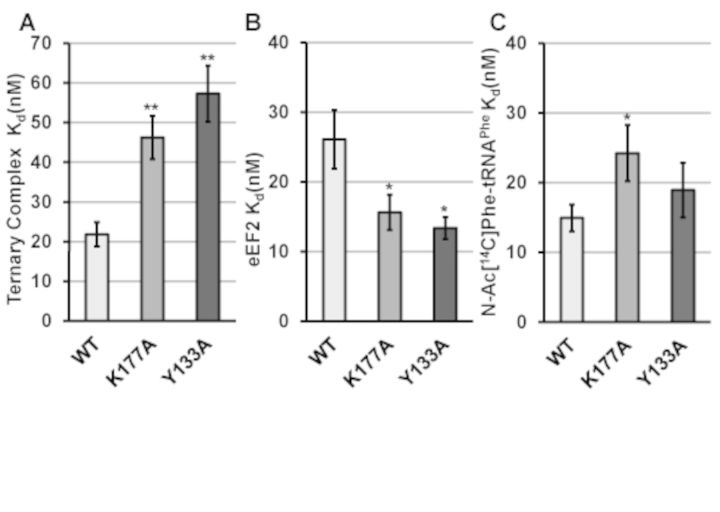
uL2 B7b bridge mutants alter the binding of translation elongation factors. Dissociation constants (K_D_) were generated by analysis of single-site binding isotherms of [^14^C]Phe-tRNA^Phe^·eEF1A·GTP (ternary complex) (**A**), or of eEF2 to the ribosomal A-site (**B**), and Ac-[^14^C]Phe-tRNA^Phe^ to the P-site (**C**). Error bars indicate standard error (n = 4, *P < 0.05, **P < 0.01).

Structural analyses of the translation elongation cycle suggested that ribosome rotational status determines the affinity of the two elongation factors with the ribosomal A-site; the TC that delivers aminoacylated tRNAs and the translocase (46). In yeast ribosomes, this model was supported using mutants of uL16, i.e. unrotated ribosomes had higher affinity for TC (eEF1A•aminoacyl-tRNA•GTP) and rotated ribosomes had higher affinity for translocase (eEF2•GTP) (16). To test the universality of this model, steady-state filter-binding assays were performed using the uL2 mutants identified in the current study. Ribosomes containing the two most rotated mutants, uL2-K177A and uL2-Y133A displayed more than 2-fold increases in K_D_ for TC (*P* < 0.01), and conversely, nearly 2-fold decreases in K_D_ for eEF2•GDPNP (*P* < 0.06) (Figure 2A, B, Supplementary Figure S4A–S4D). uL2–248–254Δ mutant ribosomes, which promoted a lesser extent of shift in rotational equilibrium, also promoted significantly higher K_D_ for TC, while uL2-H139-E143A, having the least pronounced effect on rotational equilibrium, did not significantly affect TC binding. The uL2-K177A and C-terminal deletion mutants also conferred higher K_D_ for N-acetyl-aminoacyl-tRNA (Ac-aa-tRNA) to the P-site, while the uL2-H139-E143A mutant promoted a small but significant increase in this parameter (Figure 2C, Supplementary Figure S4E, F).

### The uL2-K177A and uL2-Y133A mutants promote significant changes in translational fidelity

**Figure 3.**
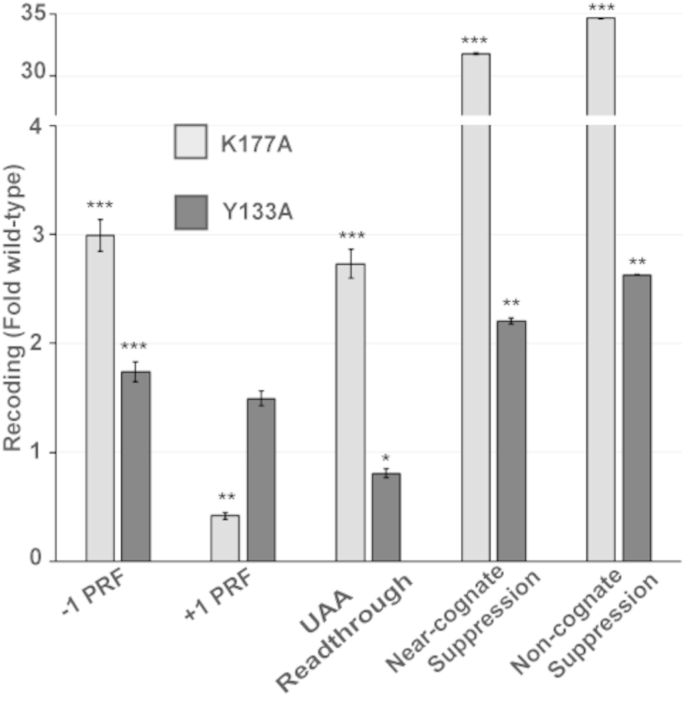
uL2-K177A and uL2-Y133A mutants promote defects in translational fidelity. Isogenic yeast cells expressing either wild-type or indicated mutant forms of uL2 were transformed with dual luciferase reporters and control plasmids and rates of translational recoding were determined. All results are graphed as fold wild-type. −1 PRF was measured using the yeast L-A virus frameshift signal. +1 PRF was directed by the frameshift signal derived from the Ty*1* retrotransposable element. Rates of termination codon readthrough were measured using a reporter harboring an in-frame UAA termination codon positioned between the *Renilla* and firefly luciferase reporter genes. Rates of suppression of missense suppression near- and noncognate codons were evaluated by incorporation of an arginine (AGA) instead of a cognate serine (AGC) or a noncognate serine (TCT) at the firefly luciferase catalytic codon 218. Error bars denote standard error. *n = 4–10* biological replicates repeated in quadruplicate, *P < 0.05, **P < 0.01.

Dual luciferase based *in vivo* assays were used to monitor the effects of the two mutants on five different aspects of translational fidelity. In general, and consistent with the strongest effects on intersubunit rotation, the uL2-K177A mutant conferred the strongest changes in translational fidelity (Figure 3) (note that these data were culled from Supplementary Figure S2C and are shown here to highlight these two mutants). Both mutants conferred increased rates of -1 PRF, consistent with studies indicating that rotated ribosomes are substrates for this reaction (47,48). In contrast, uL2-K177A ribosomes promoted a >50% decrease in rates of +1 PRF, while uL2-Y133A mutants promoted a modest but statistically insignificant increase. uL2-K177A also led to a nearly 3-fold increase in readthrough of the UAA termination codon. However, the most pronounced effects were observed in conjunction with the effects of this mutant on the ability of ribosomes to discriminate between cognate and either near-or noncognate tRNAs, where greater than 30-fold increases in suppression of these classes of codons were observed.

### The uL2-K177A and uL2-Y133A mutants affect gene expression and telomere maintenance through changes in programmed ribosomal frameshifting

**Figure 4.**
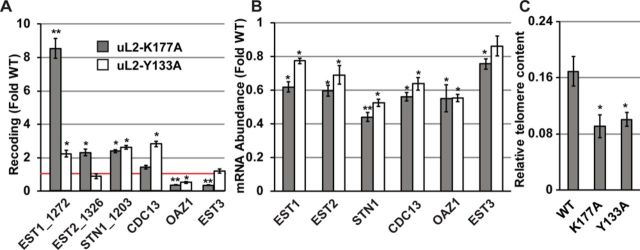
uL2-K177A and uL2-Y133A mutant ribosomes promote defects in cellular gene expression and telomere maintenance through altered frameshifting. (**A**) Rates of −1 PRF were determined by sequences derived from yeast *EST1* (signal beginning at nucleotide 1272), *EST2* (signal beginning at nucleotide 1326), *STN1* (signal beginning at nucleotide 1203) and *CDC13* (signal beginning at nucleotide 1272) and of +1 PRF directed by sequence in the *OAZ1* and *EST3* mRNAs. (**B**) Steady-state abundance of endogenous EST1, EST2, STN1, CDC13, OAZ1 and EST3 mRNAs was monitored by quantitative RT-PCR. Bars indicate SEM. (**C**) The abundance of telomere repeat sequences was quantified by PCR, with the single-copy reference gene SGS1 as the loading control. T/S ratios calculated from C_t_ values represent relative telomere content. Bars indicate SEM (n = 9). *P < 0.05; **P < 0.01.

In yeast, the mRNAs encoding at least four proteins (EST1, EST2, STN1, CDC13) involved in telomere maintenance contain -1 PRF signals that function as destabilizing elements by directing translating ribosomes to premature termination codons (17). The EST3 mRNA encoding a fifth telomere maintenance protein harbors a +1 PRF signal that is identical to the Ty*1* recoding element that is required for synthesis of the full-length protein (49,50). Additionally, OAZ1, encoding ornithine decarboxylase antizyme requires a +1 PRF event for its synthesis (51). Dual luciferase reporters harboring these frameshift signals were assayed in isogenic wild-type, uL2-K177A and uL2-Y133A cells (Figure 4A). These assays revealed that -1 PRF directed by signals in the EST1, EST2 and STN1 mRNAs was stimulated in the uL2-K177A mutant, while -1 PRF directed by the CDC13 sequence was only slightly stimulated by this mutation. In general, the uL2-Y133A mutant also stimulated -1 PRF directed by these signals with slight variations. +1 PRF directed by the OAZ1 sequence was also strongly inhibited by both mutants (<25–40% of wild-type). qRT-PCR assays revealed decreases in the steady-state abundances of all of the PRF signal containing mRNAs in both mutants (Figure 4B). Decreased abundance of the -1 PRF signal containing mRNAs is consistent with findings that they function as mRNA destabilizing elements by directing translating ribosomes to premature termination codons (17,52). Similarly, since +1 PRF events are required for synthesis of full-length Est3p and Oaz1p, decreased rates of +1 PRF are predicted to increase the fraction of ribosomes directed to premature termination codons on these mRNAs, also reducing their steady-state abundances. qRT-PCR assays revealed that the abundance of telomere DNA repeats (a proxy for telomere length) in both uL2-K177A and uL2-Y133A mutant cells was reduced approximately 2-fold compared to isogenic wild-type cells (Figure 4C). This is consistent with the idea that PRF plays an important role in yeast telomere maintenance.

## DISCUSSION

**Figure 5.**
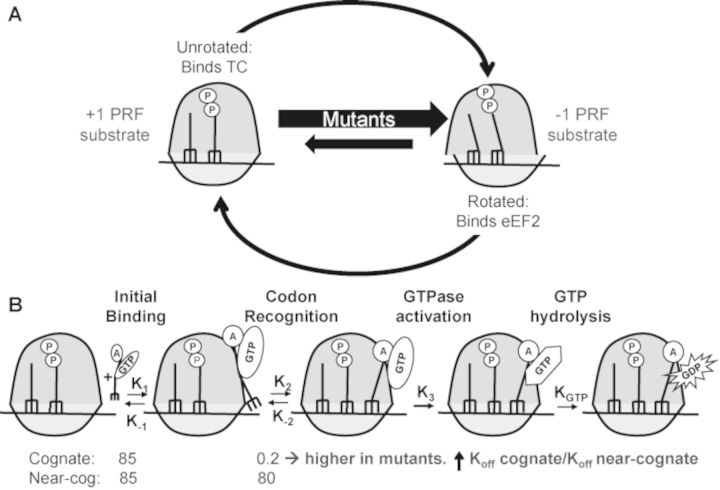
Model describing the effects of disturbed ribosomal rotational equilibrium on tRNA selection and translational fidelity. (**A**) The uL2 B7b bridge mutants perturb the dynamic rotational equilibrium of ribosomes by shifting it toward the rotated state. This increases the steady-state abundance of -1PRF substrate (rotated ribosomes) while creating a deficit of +1 PRF substrate (unrotated ribosomes). (**B**) Kinetics of initial selection and ternary complex binding is affected by uL2 mutants. In wild-type ribosomes, lower K_off_ rates for cognate ternary complex than the near-cognate normally ensure high fidelity of tRNA selection (∼400-fold) (78). Shifting rotational equilibrium toward the rotated state disproportionately increases the K_off_ for cognate ternary complex relative to near- or noncognate (which are already fast). This results in decreased selectivity at the codon recognition step of translation elongation.

The ribosome transits through a large number of conformational states during the translation elongation cycle, the two extremes of which are called unrotated and rotated (8–9,53). The structural and biochemical analyses with the uL2 mutants (Figures 1 and 2) demonstrate a direct relationship between ribosome rotational status and affinity for the two elongation factors. These observations broaden the support for the general model in which unrotated ribosomes have higher affinity for TC and lower affinity for the translocase, and these properties are reversed upon intersubunit rotation (Figure 5A). Previous kinetics studies suggested the presence of at least two conformations of the empty ribosomes responsible for preferential binding of either EF-Tu·GTP or EF-G·GTP (54). These conformational states were not called rotated or unrotated but it was suggested that the empty ribosomes oscillate between the two states.

The selection of appropriate ligands to the ribosomal A-site during translation elongation is governed by a delicate series of kinetic balances (55–57). Disruption of these parameters by the uL2 mutants examined here is manifested at the level of rRNA structure by the observed changes in rRNA chemical modification patterns. The demonstration of changes in rRNA structure in all of the functional centers located in both subunits is consistent with allosteric conformational changes identified using other mutants of yeast proteins and rRNA bases (16,28,58–64). Increased reactivity of bases in the B7a intersubunit bridge is diagnostic of the intersubunit rotational equilibrium having shifted toward the rotated state. This is further supported by the observed closing of the tRNA accommodation corridor (decreased reactivity in H89–H92), stabilization of A2971 (*E. coli* 2602) in the peptidyltransferase center, and stabilization of h27. These observations lend further support to the model linking ribosome rotational status to elongation factor binding affinity. Here, we present a model illustrating how these changes in structure and biochemistry account for the observed changes in translational fidelity (Figure 5). The substrate for Ty*1* and EST3 directed +1 PRF is a ribosome paused at the CUU AGG C heptameric slippery site with a peptidyl-tRNA base paired to the P-site CUU codon, and awaiting the rare cognate tRNA(CCU-Arg) at the A-site (65), i.e. an unrotated ribosome (Figure 5a). In contrast, rotated ribosomes containing hybrid-state tRNAs have been demonstrated to be the substrates for -1 PRF (47–48,66–67) (Figure 5a). By shifting the rotational equilibrium toward the rotated state, these mutants increase the steady-state abundance of -1 PRF substrate, thus stimulating this process. In contrast, decreased abundance of the +1 PRF substrate results in decreased rates of +1 PRF. Similarly, since the release factors bind to unrotated ribosomes, decreasing this substrate inhibits this function, leading to increased rates of UAA termination codon readthrough, at least for the uL2-K177A mutant (i.e. the mutant that most disrupted rotational equilibrium). The most dramatic changes in translational fidelity were observed as defects in the ability of ribosomes to discriminate between cognate tRNAs and near-/noncognate tRNAs. As shown in Figure 5b, the unrotated ribosomes are the substrate for TC binding. Elegant biochemical studies have detailed the kinetic parameters underlying the high fidelity selection of cognate tRNAs during the translation elongation process (56,68,78). Importantly, these analyses have shown that rapid dissociation of near-cognate tRNAs from ribosomes is the first step of discrimination. Specifically, near-cognate tRNAs dissociate at rates of ∼80 s^−1^ as compared to ∼0.2 s^−1^ for cognate tRNAs, a 400-fold difference. However, shifting the population of ribosomes toward the rotated state increases the steady-state K_D_ of ribosomes for all TCs. We suggest that this disproportionately affects TCs harboring cognate tRNAs, reducing ability of ribosomes to discriminate between cognate and near-cognate TCs. We note that the model shown in Figure 5 is simplistic and that the full picture is undoubtedly much more complex. For example, there is more conformational complexity to the ribosome than simply ‘unrotated’ and ‘rotated’. Indeed, it is estimated that the ribosome transits through approximately 50 conformational states (12). The hSHAPE analyses employed in the current study cannot distinguish among all of these, and it is quite possible that the two mutants have differential effects on this complex set of equilibria that may manifest as differences in ligand binding and ultimately, translational fidelity. Additionally, the two mutants may confer different subtle changes in local rRNA structures that may also affect ligand binding and translational fidelity. The strength of this model is that it illuminates a general principle that will serve as a guide to more fine-grained research in the future.

Expression of up to 10% of cellular genes is predicted to be regulated by -1 PRF (69,70), and +1 PRF has been demonstrated in at least two additional yeast mRNAs (71,72). -1 PRF events in yeast mRNAs encoding proteins critical for telomere maintenance direct translating ribosomes to premature termination codons, resulting in mRNA degradation through the nonsense-mediated mRNA decay pathway (NMD) (17,52). Thus, increased rates of -1 PRF promoted by the uL2-K177A and uL2-Y133A mutants promoted decreased abundances of the -1 PRF signal containing EST1, EST2, STN1 and CDC13 mRNAs. In contrast, +1 PRF is required to complete translation of the EST3 and OAZ1 mRNAs. Here, decreased rates of +1 PRF results in a greater fraction of ribosomes encountering the 0-frame termination codons in these mRNAs, decreasing their abundances presumably also through NMD. At the biological level, the proteins encoded by *EST* family of genes, *STN1* and *CDC13* are all involved in yeast telomere maintenance (73). The observed changes in -1 and +1 PRF resulted in decreased abundance of yeast telomere repeats, indicative of enhanced telomere shortening. While it is not known if mRNAs encoding proteins required for human telomere maintenance are regulated by PRF, should this be demonstrated in the future, the findings presented here may provide a link between PRF and the progeria-like presentations of some ribosomopathies (74). *OAZ1* encodes ornithine decarboxylase antizyme, the critical control node in polyamine biosynthesis (75). Disruption of this pathway has pleiotropic effects, including control of cellular proliferation and development (76,77). The recent demonstration of -1 PRF and termination codon readthrough regulated gene expression humans provide evidence for importance of translational recoding in fine-tuning the immune system and control of tumor progression, respectively (18,19). The effects of the uL2 mutants on translational fidelity described in this study may be helpful in deepening our understanding the complex nature of cancer progression.

## SUPPLEMENTARY DATA

Supplementary Data are available at NAR Online.

SUPPLEMENTARY DATA
