# Supplementary material for: Ribosomes in the balance: structural equilibrium ensures translational fidelity and proper gene expression
Source: Nucleic Acids Res. 2014 Nov 11;42(21):13384–92. doi: 10.1093/nar/gku1020 (PMC4245932; doi:10.1093/nar/gku1020)
Supplement: SUPPLEMENTARY DATA [file supp_42_21_13384__index.html]

Ribosomes in the balance: structural equilibrium ensures translational fidelity and proper gene expression — Ribosomes in the balance: structural equilibrium ensures translational fidelity and proper gene expression — SUPPLEMENTARY DATA 

# Ribosomes in the balance: structural equilibrium ensures translational fidelity and proper gene expression

## SUPPLEMENTARY DATA

**Files in this Data Supplement:**

- SUPPLEMENTARY DATA
