# Supplementary material for: Ribosomes in the balance: structural equilibrium ensures translational fidelity and proper gene expression
Source: Nucleic Acids Res. 2014 Nov 11;42(21):13384–92. doi: 10.1093/nar/gku1020 (PMC4245932; doi:10.1093/nar/gku1020)
Supplement: SUPPLEMENTARY DATA [file supp_gku1020_nar-02035-v-2014-File007.docx]

**Supplementary Data**

**Supplementary Table 1.** Strains of *S. cerevisiae* generated and used in this study

| **Strain** | **Genotype** |
| --- | --- |
| 5X47 | *MATa/MATa his1/+ trp1/+ ura3/+* K^-^ |
| JD1269 | *MATa his3D1 leu2D0 met15D0 ura3D0 rpl2a::KAN^r^ rpl2b::KAN^r^* [L-A HN M_1_] pRPL2A-URA3 |
| JD1315 | *MATa his3D1 leu2D0 met15D0 ura3D0 rpl2a::KAN^r^ rpl2b::KAN^r^* [L-A HN M_1_] pRPL2A-LEU2 |
| JD1676 | *MATa his3D1 leu2D0 met15D0 ura3D0 rpl2a::KAN^r^ rpl2b::KAN^r^* [L-A HN M_1_] pRPL2A(H139-E143A)-LEU2 |
| JD1677 | *MATa his3D1 leu2D0 met15D0 ura3D0 rpl2a::KAN^r^ rpl2b::KAN^r^* [L-A HN M_1_] pRPL2A(D176A)-LEU2 |
| JD1678 | *MATa his3D1 leu2D0 met15D0 ura3D0 rpl2a::KAN^r^ rpl2b::KAN^r^* [L-A HN M_1_] pRPL2A(K177A)-LEU2 |
| JD1679 | *MATa his3D1 leu2D0 met15D0 ura3D0 rpl2a::KAN^r^ rpl2b::KAN^r^* [L-A HN M_1_] pRPL2A(V148A)-LEU2 |
| JD1680 | *MATa his3D1 leu2D0 met15D0 ura3D0 rpl2a::KAN^r^ rpl2b::KAN^r^* [L-A HN M_1_] pRPL2A(G138A)-LEU2 |
| JD1681 | *MATa his3D1 leu2D0 met15D0 ura3D0 rpl2a::KAN^r^ rpl2b::KAN^r^* [L-A HN M_1_] pRPL2A(L245-S249A)-LEU2 |
| JD1682 | *MATa his3D1 leu2D0 met15D0 ura3D0 rpl2a::KAN^r^ rpl2b::KAN^r^* [L-A HN M_1_] pRPL2A(H139-P141A)-LEU2 |
| JD1683 | *MATa his3D1 leu2D0 met15D0 ura3D0 rpl2a::KAN^r^ rpl2b::KAN^r^* [L-A HN M_1_] pRPL2A(D142-E143A)-LEU2 |
| JD1684 | *MATa his3D1 leu2D0 met15D0 ura3D0 rpl2a::KAN^r^ rpl2b::KAN^r^* [L-A HN M_1_] pRPL2A(P108-E109A)-LEU2 |
| JD1685 | *MATa his3D1 leu2D0 met15D0 ura3D0 rpl2a::KAN^r^ rpl2b::KAN^r^* [L-A HN M_1_] pRPL2A(G110-T111A)-LEU2 |
| JD1686 | *MATa his3D1 leu2D0 met15D0 ura3D0 rpl2a::KAN^r^ rpl2b::KAN^r^* [L-A HN M_1_] pRPL2A(K155A)-LEU2 |
| JD1687 | *MATa his3D1 leu2D0 met15D0 ura3D0 rpl2a::KAN^r^ rpl2b::KAN^r^* [L-A HN M_1_] pRPL2A(G248-K254A)-LEU2 |
| JD1688 | *MATa his3D1 leu2D0 met15D0 ura3D0 rpl2a::KAN^r^ rpl2b::KAN^r^* [L-A HN M_1_] pRPL2A(248-254Δ)-LEU2 |
| JD1689 | *MATa his3D1 leu2D0 met15D0 ura3D0 rpl2a::KAN^r^ rpl2b::KAN^r^* [L-A HN M_1_] pRPL2A(V145A)-LEU2 |
| JD1690 | *MATa his3D1 leu2D0 met15D0 ura3D0 rpl2a::KAN^r^ rpl2b::KAN^r^* [L-A HN M_1_] pRPL2A(R147A)-LEU2 |
| JD1691 | *MATa his3D1 leu2D0 met15D0 ura3D0 rpl2a::KAN^r^ rpl2b::KAN^r^* [L-A HN M_1_] pRPL2A(R147-V148A)-LEU2 |
| JD1692 | *MATa his3D1 leu2D0 met15D0 ura3D0 rpl2a::KAN^r^ rpl2b::KAN^r^* [L-A HN M_1_] pRPL2A(Y133A)-LEU2 |

**Supplementary Table 2.** Synthetic oligonucleotides used in this study

|  | Oligo name | Sequence |
| --- | --- | --- |
| 1 | L2A-5'UTR(FOR) | 5’CCCTGCCCCTCCCCTCCTTCAATATCATTACCTCG3’ |
| 2 | L2A-3UTR(REV) | 5’GCCATTTTATTCAAGAAGTCAACCCCCTCCATGAAGCAATGCTT3’ |
| 3 | L2A-P108-T111A(REV) | 5’GGAGACAATagcagcagcagcGACAGAACCC3’ |
| 4 | L2A-H139-E143A(REV) | 5’GTCTTGTTagcagcagcagcagcACCGATG3’ |
| 5 | L2A-S152-K155A(FOR) | 5’GATTACCAgctgctgctgctAAGGTTATC3’ |
| 6 | L2A-D176-K177A(FOR) | 5’GGTGGTAGAGTTgctgctCCATTGTTGAAGG3’ |
| 7 | L2A-R241-G244A(FOR) | 5’GCCGCCgctgctgctgctTTGTTACGTGG3’ |
| 8 | L2A-D176A(FOR) | 5’GCCGGTGGTGGTAGAGTTgctAAACCATTGTTGAAGGC3’ |
| 9 | L2A-K177A(FOR) | 5’GCCGGTGGTGGTAGAGTTGACgctCCATTGTTGAAGGC3’ |
| 10 | L2A-V148A(FOR) | 5’CGAAAACAAGACTAGAgctAGATTACCATCCGGTGCC3’ |
| 11 | L2A-G138A(REV) | 5’CGTCTGGGTTGTGagcGATGATAATAACGTAGTTACCGG3’ |
| 12 | L2A-L245-S249A(REV) | 5’ATAATACTAGTCTAagcagcagcagcagcAGAACCACGTAACA3’ |
| 13 | L2A-H139-P141A(REV) | 5’CTCTAGTCTTGTTTTCGTCagcagcagcACCGATGATAATAACG3’ |
| 14 | L2A-D142-E143A(REV) | 5’CTGACTCTAGTCTTGTTagcagcTGGGTTGTGACCGATGATA3’ |
| 15 | L2A-P108-E109A(REV) | 5’GGAGACAATagcagcTTCTGGGACAGAACCC3’ |
| 16 | L2A-G110-T111A(REV) | 5’GGAGACAATGGTACCagcagcGACAGAACCC3’ |
| 17 | L2A-S152-G153A(FOR) | 5’GATTACCAgctgctGCCAAGAAGGTTATC3’ |
| 18 | L2A-K155A(FOR) | 5’GATTACCATCCGGTgctgctAAGGTTATC3’ |
| 19 | L2A-G248-K251A(REV) | 5’CTAATCTTGGGTagcagcagcagcACGTAACAAACC3’ |
| 20 | L2A-G248-D254Δ(REV) | 5’GACTATTTTACTAGTACATAATctacaaaccagttcttctggc3’ |
| 21 | L2A-V175A(FOR) | 5’GCCGGTGGTGGTAGAgctGACAAACCATTGTTGAAGGC3’ |
| 22 | L2A-R147A(FOR) | 5’GACGAAAACAAGACTgctGTCAGATTACCATCCGG3’ |
| 23 | L2A-R147-V148A(FOR) | 5’GACGAAAACAAGACTgctgctAGATTACCATCCGG3’ |
| 24 | L2A-Y133A(FOR) | 5’GCCAGAGCTTCCGGTAACgctGTTATTATCATCGG3’ |

**Figure S1**. **Location of uL2 within the yeast ribosome**: **A.** **View of the ribosome from the E-site.** 18S and 25S rRNA is shown in grey, 5S and 5.8S rRNA in cyan. uL2 is shown in blue, peptidyltransferase center in red and decoding center in green. uL2 makes the intersubunit Bridge B7b interacting with helices h23 and h24 (shown in red) of SSU. **B. Crown view of the large subunit** shows strategic localization of ribosomal proteins near the peptidyltransferase center. Finger like basic insertion of uL2 closely approaches the peptidyltransferase center (PTC) while the acidic globular domains make contact with the small subunit through the B7b Bridge. List of mutants of *rpL2A* ORF generated in this study and their location on uL2 (**C and D).** Color codes are used to highlight the general regions mutagenized in uL2.Ribosomal structures generated in PyMol using 3Å resolution yeast ribosomal structures(1)

**Figure S2. *rpL2A* bridge mutants promote various phenotypic defects. A.** Ten-fold serial dilutions of cultures of indicated *S.cerevisiae* strains were spotted on rich medium and incubated for 48 hours at 30°C, 15°C, and 37°C to score for growth, cold and heat sensitivity respectively. **B. “Killer” virus phenotypes**: The Killer+ phenotype is scored by the presence of a halo of growth inhibition around wild-type colony. Lack of the halo around colonies expressing the H139-E143A and K177A uL2 mutants indicates the Killer- phenotype. Mutant Y133A displays a weak killer phenotype. **C. rpL2A mutants promote specific defects in translational fidelity.** Isogenic yeast cells expressing either wild-type or mutant forms of uL2 were transformed with the dual luciferase reporter and control plasmids and control plasmids and rates of translational recoding were determined. All results are graphed as fold wild type. −1 PRF was measured using the yeast L-A virus frameshift signal. +1 PRF was directed by the frameshift signal derived from the Ty1 retrotransposable element. UAA suppression denotes the percentage of ribosomes able to suppress an in-frame UAA termination codon positioned between the Renilla and firefly luciferase reporter genes. Missense suppression rates were evaluated by incorporation of an arginine (AGA) near-cognate amino acid instead of a cognate serine (AGC) at the catalytic codon 218 within the firefly luciferase gene. Error bars denote standard error calculated as described in (2).

**Figure S3.Structural probing analysis of Wild-type and uL2-K177A:** Chemical probing analysis using 1M7 and hSHAPE was performed as described in (3), Reactivity difference between uL2-K177A and WT for the bases covered was mapped on the 2d map of 18S rRNA (**A**) and 25S rRNA (**B**). The scale at right indicates the extent of differences in reactivities with each number corresponding to one standard deviation from the mean reactivity as previously described (3). Magnified view of h23 and h24 (harbor B7b bridge forming residues) in **A**, displaying difference in reactivity between WT and uL2-K177A. Change in flexibility seen in h27 important in translational fidelity. In 25S rRNA (**B**), difference in IM7 reactivity were seen in bases in peptidyltransferase center, H93, and H69. Heat maps were generated by applying the color-coded SHAPE reactivity data to the 3d-based maps and Ribovision:Version 1.15 (4).

**Figure S4. A.** Single site binding isotherms of ternary complex to the A-site of ribosomes isolated from wild-type, *rpl2- H139-E143A, rpl2-K177A, rpl2-G248-D254Δ* and *rpl2-Y133A* cells (panel a). **B**. Ternary complex binding dissociation constants (K_d_s) calculated using ligand depletion model (Graphpad Prizm). **C**. Single site binding isotherms of eEF2 to ribosomes isolated from wild-type, *rpl12-K177A,* and *rpl2-Y133A* cells. **D.** eEF2 binding K_d_s calculated using ligand depletion model (Graphpad Prizm). **E**. Single site binding isotherms N-Ac-Phe-tRNA^Phe^ to the P-site of ribosomes isolated from wild-type, *rpl2- H139-E143A, rpl2-K177A, rpl2-G248-D254Δ* and *rpl2-Y133A* cells. **F**. N-Ac-Phe-tRNA^Phe^ binding K_d_s calculated using ligand depletion model (Graphpad Prizm). Error bars indicate standard error. (n=4, * *P* < 0.06, ** P<.001)

1. Ben Shem, A., Garreau, de L., Melnikov, S., Jenner, L., Yusupova, G. and Yusupov, M. (2011) The structure of the eukaryotic ribosome at 3.0 A resolution. *Science (80-. ).*, **334**, 1524–1529.

2. Jacobs, J.L. and Dinman, J.D. (2004) Systematic analysis of bicistronic reporter assay data. *Nucleic Acids Res.*, **32**, e160.

3. Leshin, J.A., Heselpoth, R., Belew, A.T. and Dinman, J.D. (2011) High throughput structural analysis of yeast ribosomes using hSHAPE. *RNA Biol.*, **8**.

4. Petrov, A.S., Bernier, C.R., Gulen, B., Waterbury, C.C., Hershkovits, E., Hsiao, C., Harvey, S.C., Hud, N. V, Fox, G.E., Wartell, R.M., et al. (2014) Secondary Structures of rRNAs from All Three Domains of Life. *PLoS One*, **9**, e88222.

**Figure S1.**

**Figure S2**


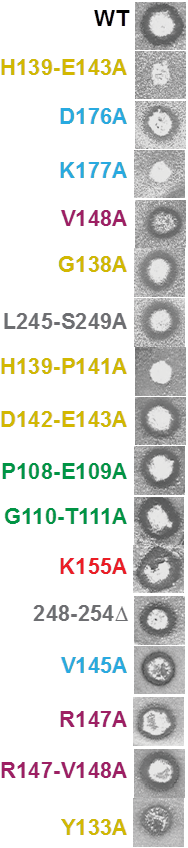


**B**

**A**


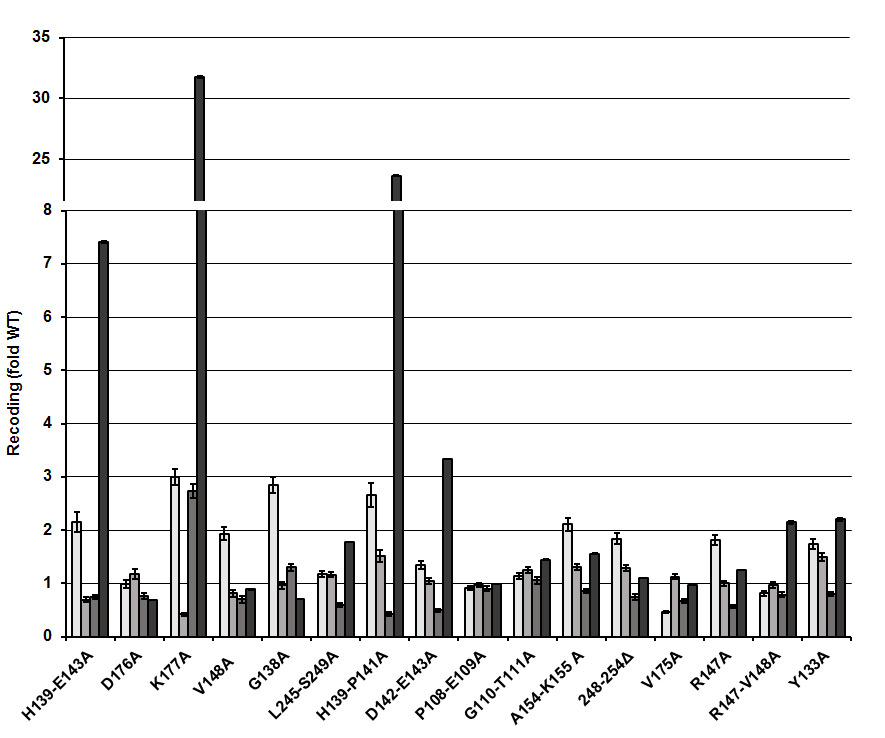


**-1 PRF (L-A)**

**+1 PRF (Ty*1*)**

**UAA Suppression**

**Near-cognate missense suppression**

**C**

**Figure S3A**


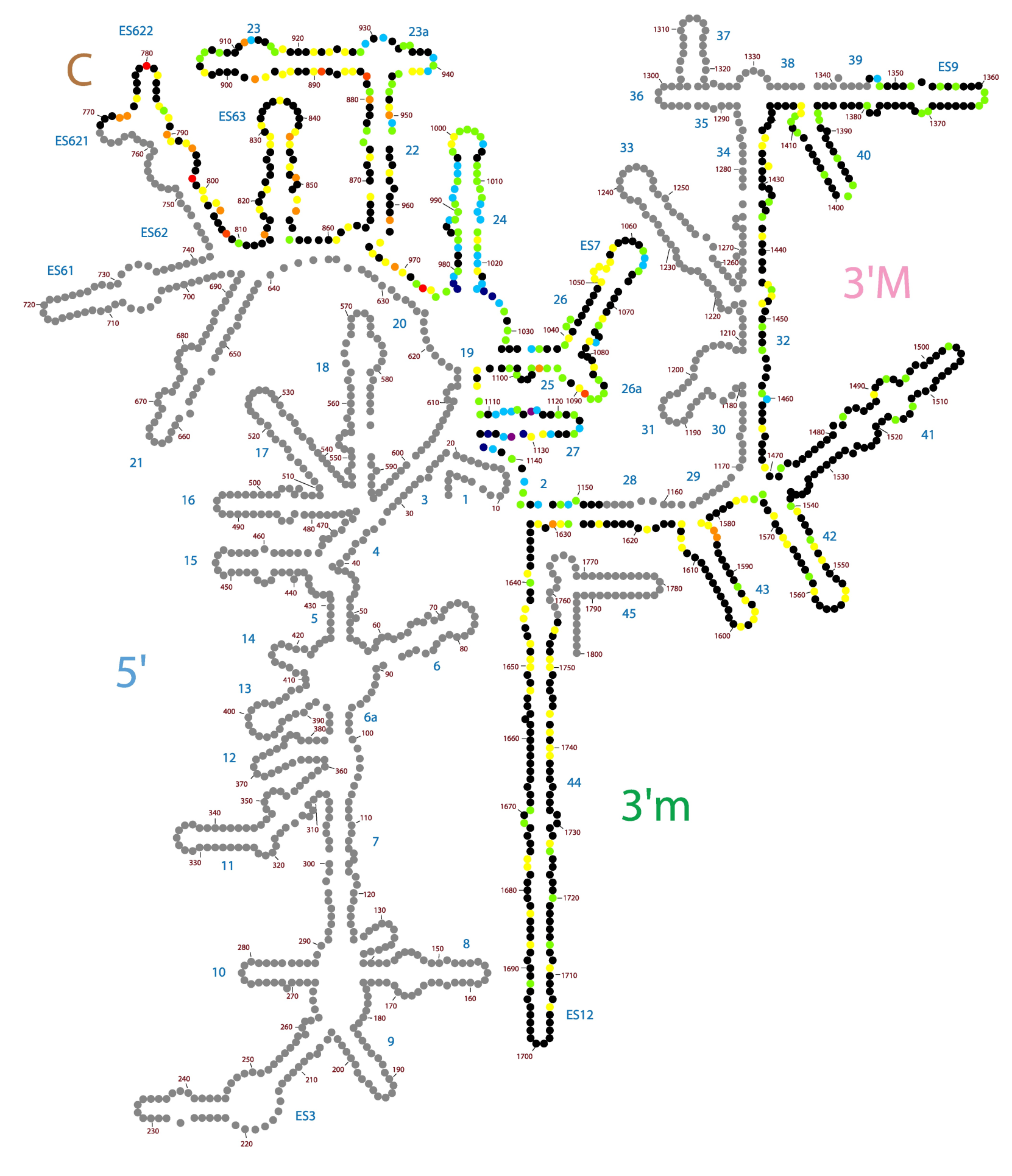

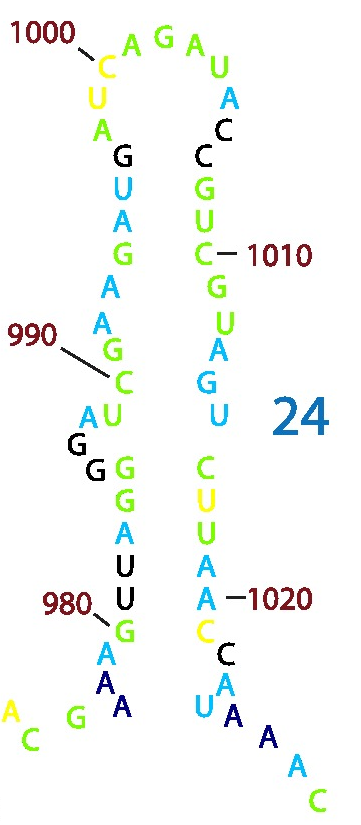

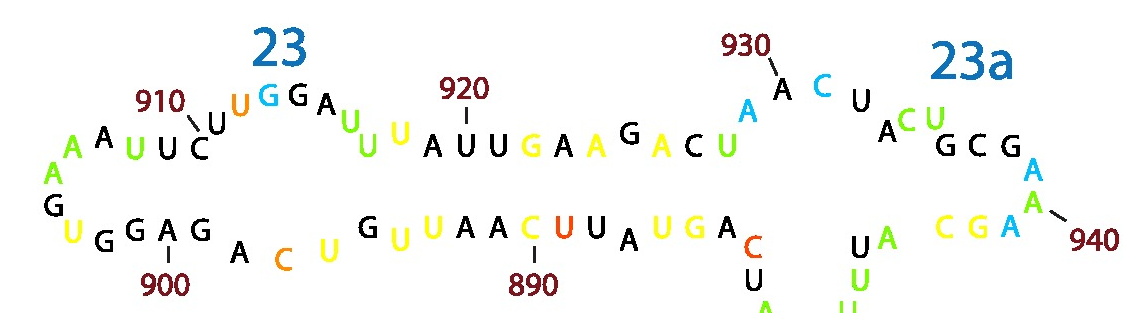


**+4**

**+3**

**+2**

**+1**

**0**

**-1**

**-2**

**-3**

**-4**

**NA**


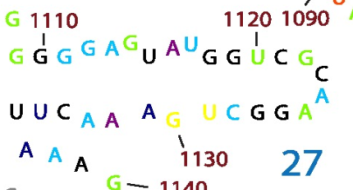


*

**Figure S3B**

**
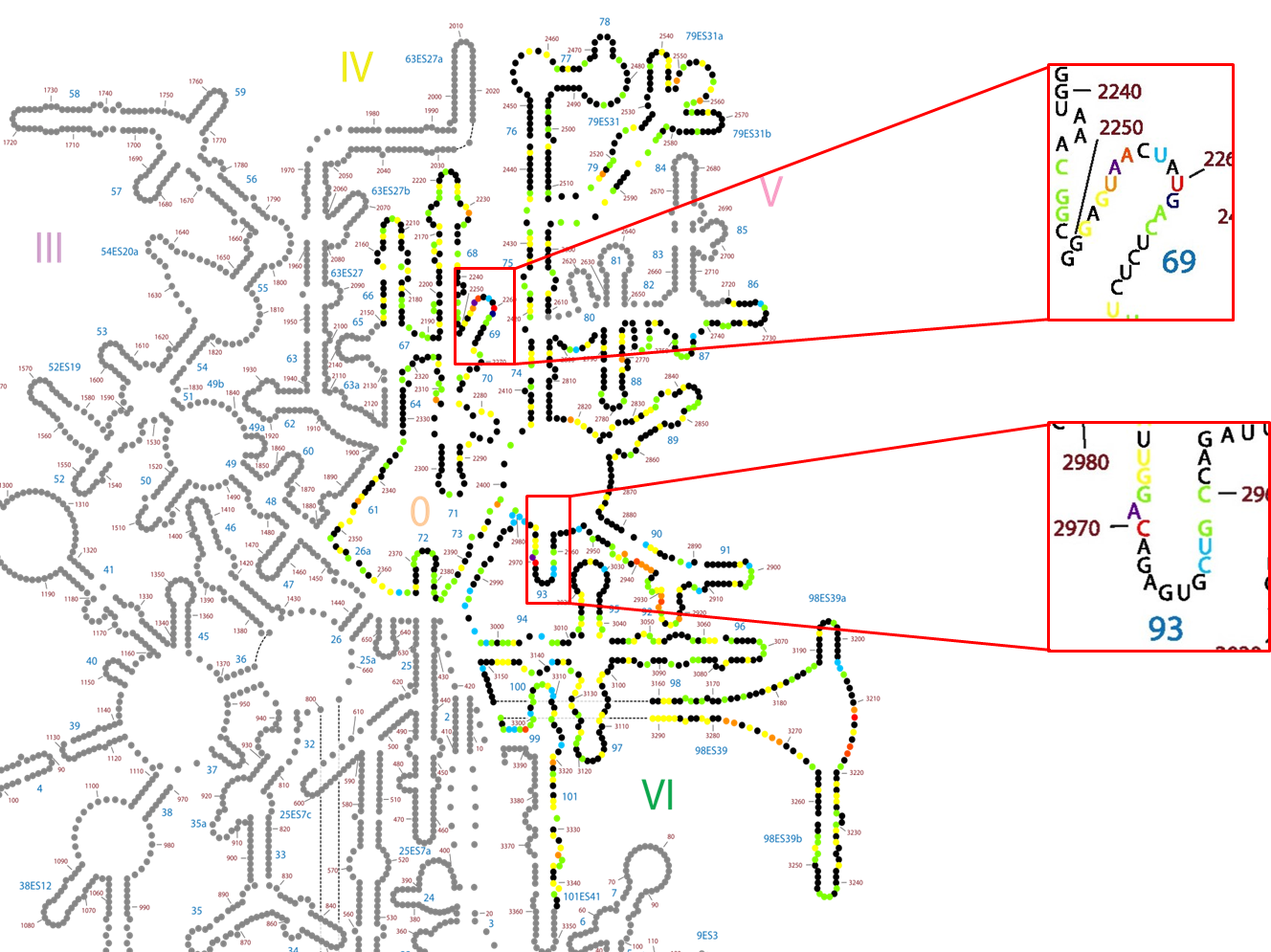
**

**Figure S4**

**
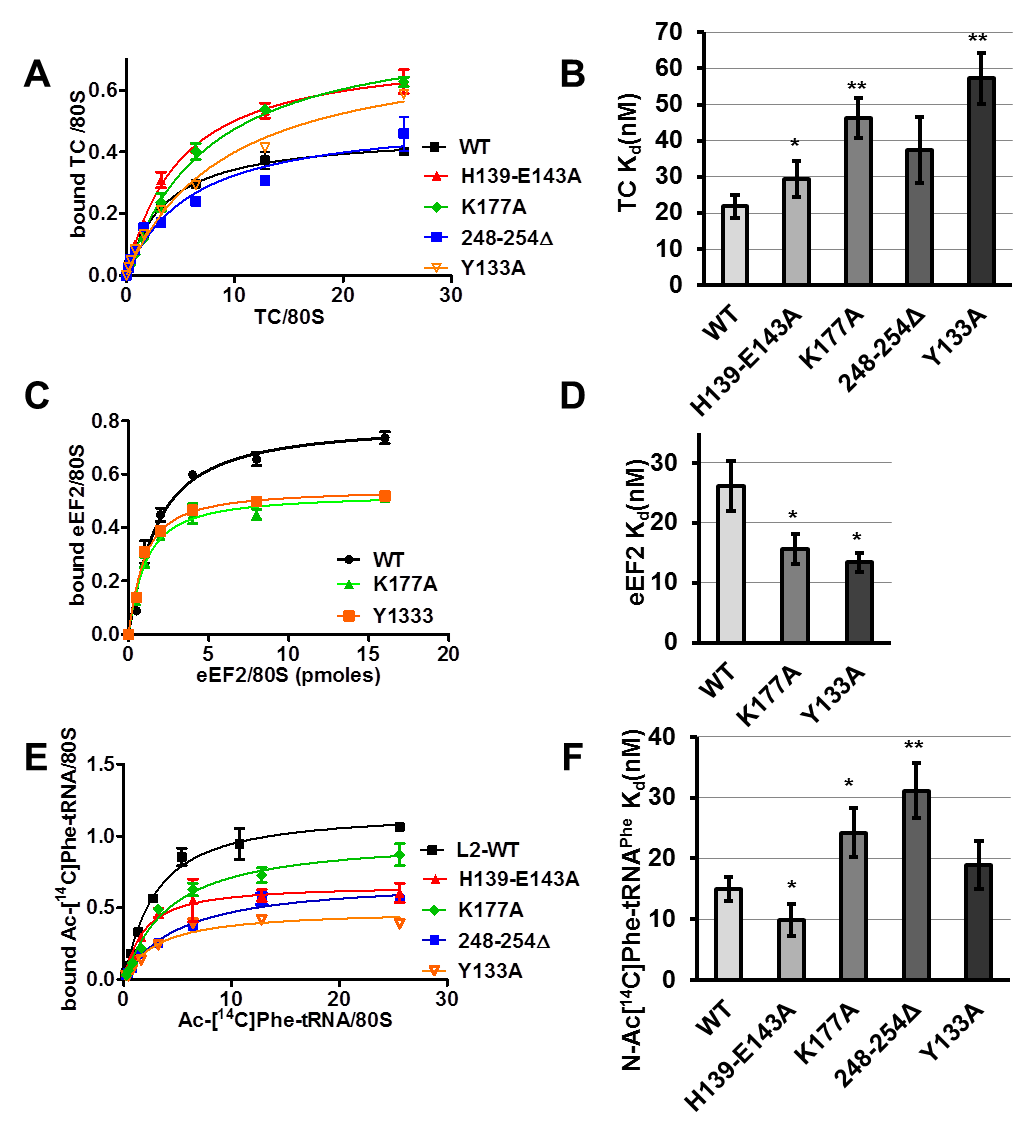
**
